# Fenofibrate Decreases Ethanol-Induced Neuroinflammation and Oxidative Stress and Reduces Alcohol Relapse in Rats by a PPAR-α-Dependent Mechanism

**DOI:** 10.3390/antiox12091758

**Published:** 2023-09-13

**Authors:** Cristina Ibáñez, Tirso Acuña, María Elena Quintanilla, Diliana Pérez-Reytor, Paola Morales, Eduardo Karahanian

**Affiliations:** 1 Institute of Biomedical Sciences, Faculty of Health Sciences, Universidad Autónoma de Chile, Santiago 8910060, Chile; cristina.ibanez@cloud.uautonoma.cl (C.I.); d.perez@uautonoma.cl (D.P.-R.); 2 Research Center for the Development of Novel Therapeutic Alternatives for Alcohol Use Disorders, Santiago 8910060, Chile; equintanilla@uchile.cl; 3 Molecular and Clinical Pharmacology Program, Institute of Biomedical Sciences, Faculty of Medicine, Universidad de Chile, Santiago 8380453, Chile; tirsoacuna@ug.uchile.cl; 4 Department of Neuroscience, Faculty of Medicine, Universidad de Chile, Santiago 8380453, Chile

**Keywords:** alcohol use disorder, alcoholism, fibrates, neuroinflammation, PPAR alpha

## Abstract

High ethanol consumption triggers neuroinflammation, implicated in sustaining chronic alcohol use. This inflammation boosts glutamate, prompting dopamine release in reward centers, driving prolonged drinking and relapse. Fibrate drugs, activating peroxisome proliferator-activated receptor alpha (PPAR-α), counteract neuroinflammation in other contexts, prompting investigation into their impact on ethanol-induced inflammation. Here, we studied, in UChB drinker rats, whether the administration of fenofibrate in the withdrawal stage after chronic ethanol consumption reduces voluntary intake when alcohol is offered again to the animals (relapse-type drinking). Furthermore, we determined if fenofibrate was able to decrease ethanol-induced neuroinflammation and oxidative stress in the brain. Animals treated with fenofibrate decreased alcohol consumption by 80% during post-abstinence relapse. Furthermore, fenofibrate decreased the expression of the proinflammatory cytokines tumor necrosis factor-alpha (TNF-α) and interleukins IL-1β and IL-6, and of an oxidative stress-induced gene (heme oxygenase-1), in the hippocampus, nucleus accumbens, and prefrontal cortex. Animals treated with fenofibrate showed an increase M2-type microglia (with anti-inflammatory proprieties) and a decrease in phagocytic microglia in the hippocampus. A PPAR-α antagonist (GW6471) abrogated the effects of fenofibrate, indicating that they are dependent on PPAR-α activation. These findings highlight the potential of fenofibrate, an FDA-approved dyslipidemia medication, as a supplementary approach to alleviating relapse severity in individuals with alcohol use disorder (AUD) during withdrawal.

## 1. Introduction

Alcohol use disorder (AUD) remains a significant public health concern, with available pharmacological interventions such as naltrexone and acamprosate, which primarily target reducing alcohol craving, help maintain abstinence, and reduce harmful drinking in the treatment phase of psychological dependence. However, they often fall short in providing sustained recovery, as most patients experience relapse in the short or middle term [1,2]. Throughout the detoxification phase, patients are typically prescribed benzodiazepines, such as diazepam, to alleviate symptoms of alcohol withdrawal syndrome [3]; however, their usage in cases of AUD should be limited to short durations due to the potential of substituting alcohol addiction with benzodiazepine dependence. Prolonged and excessive alcohol consumption leads to enduring changes in the brain, perpetuating addictive behavior in individuals with AUD. Ethanol-induced neuroinflammation in the brain has emerged as a pivotal factor in the neurobiological changes associated with persistent chronic alcohol abuse [4,5,6].

Neuroinflammation associated with ethanol consumption can be induced by two mechanisms: one of them involves the oxidation of ethanol by cytochrome P4502E1 (CYP2E1), generating reactive oxygen species (ROS) that activate nuclear factor kappa-light-chain-enhancer of activated B cells (NF-κB), leading to pro-inflammatory responses [7,8]. This activation also increases the expression of NADPH oxidase [9], which generates even more ROS, perpetuating the inflammatory response [5,6]. The second mechanism involves the activation of tumor necrosis factor-alpha (TNF-α) receptor in the brain, since ethanol consumption increases the permeability of the intestinal mucosa, which allows bacterial lipopolysaccharide (LPS) to diffuse into circulation [10]. LPS then triggers the release of TNF-α to the blood, which can cross the blood–brain barrier and activate microglial TNF receptors, inducing neuroinflammation [11,12]. The activation of either mechanism leads to the release of proinflammatory cytokines such as TNF-α, IL-1β, and IL-6 [13]. This glial-secreted TNF-α then binds to its TNF receptor in the same microglial cells, creating an activation loop that potentiates the initial neuroinflammation response [5]. The self-perpetuating positive feedback loops created by these mechanisms can maintain neuroinflammation for months after alcohol withdrawal, which increases the risk of relapse [9].

The establishment of alcohol addiction is a complex process, where the modulation of dopamine release in the brain’s reward pathways plays a central role. This modulation involves interactions between various neurotransmitters’ pathways, including γ-aminobutyric acid (GABA) and glutamate. Both neurotransmitters modulate dopamine release in the nucleus accumbens in response to ethanol. Activation of presynaptic GABA receptors by ethanol inhibits GABA release in the ventral tegmental area (VTA), which in turn stimulates dopamine release in the nucleus accumbens [14]. Dopamine release is closely linked to the motivational effects of alcohol, that is, to the positive reinforcement involved in the reward. However, a second behavioral aspect of AUD is negative reinforcing features such as hyper-anxious and hyperexcitability states, which lead the individual to continue drinking to obtain relief from this negative affective state associated with alcohol dependence. GABAergic mechanisms have been implicated in the neuroadaptations associated with the transition in humans from limited access to ethanol to chronic drinking [15]. Studies with pharmacological agonists and antagonists have implicated GABA systems in the anxiogenic effects of ethanol withdrawal, since GABA agonists decrease central nervous system hyperexcitability during ethanol withdrawal, whereas GABA antagonists exacerbate many of the symptoms of ethanol withdrawal [15]. Although there are no studies that demonstrate the direct relationship between ethanol-induced neuroinflammation and anxiogenic effects, studies have shown that the state of anxiety could be related to the release of proinflammatory cytokines [16]. Involvement of proinflammatory cytokines in anxiety has been demonstrated in transgenic mice lacking TNF-α receptors [17] and transgenic mice lacking IL-1β receptors [18]. In these animal models, the level of anxiety was found to be lower than that in the wild-type mice [17,18]. However, the involvement of GABAergic systems in anxiety triggered by neuroinflammation has not yet been demonstrated.

On the other hand, glutamate is a neurotransmitter strongly linked to the maintenance of chronic ethanol intake and relapse to alcohol and other substances of abuse [19]. Ethanol-induced neuroinflammation plays a pivotal role in heightening glutamatergic activity in the brain [20]. Ethanol is known to increase the extracellular glutamate levels within mesocorticolimbic structures. In AUD individuals, astrocytes exhibit modified glutamate, clearance due to the inadequate functioning of the brain’s glutamate transporter 1 (GLT-1), also recognized as excitatory amino acid transporter 2 (EAAT2) or solute carrier family 1 member 2 (SLC1A2). This irregularity results in an increase in extracellular glutamate levels at the tripartite synapse [21]. Following chronic ethanol consumption and subsequent abstinence, environmental cues associated with alcohol usage can trigger drug-seeking behavior and the urge to re-administer the substance, leading to relapse and the perpetuation of alcohol intake [22]. This behavior cycle is powered by an amplified glutamate release into the nucleus accumbens through intricate circuit connecting hippocampus and prefrontal cortex [20].

Peroxisome proliferator-activated receptor alpha (PPAR-α) is a nuclear receptor with key functions in lipid metabolism [23]. Fibrate drugs such as fenofibrate, bezafibrate, gemfibrozil, ciprofibrate, and clofibrate are PPAR-α agonists [24], actively elevating the oxidation rate of fatty acids, and are commonly employed to treat hypertriglyceridemia [25]. Several studies have shown that fenofibrate administration to rodents that consume alcohol voluntarily leads to a reduction in alcohol intake [26,27,28,29,30,31]. Since PPAR-α activation in the brain decreases neuroinflammation [32,33,34,35,36], we hypothesized that fenofibrate exerts an anti-inflammatory effect even when administered only during the abstinence period following chronic alcohol intake. In line with this hypothesis, a previous study by our group reported that the administration of fenofibrate during the abstinence period following chronic alcohol consumption in rats was able to (i) reverse the ethanol-induced increase in glial acidic fibrillary protein (GFAP) levels, indicative of astrogliosis, (ii) decrease the deactivation of the NF-κB inhibitor (IκBα), and (iii) restore the diminished expression levels of GLT-1 caused by alcohol treatment [37].

In this study, our primary objective was to examine the potential effects of fenofibrate when administered exclusively during the abstinence stage following chronic alcohol consumption in rats, specifically focusing on its impact on alcohol intake in a relapse model. Additionally, we sought to evaluate the influence of fenofibrate administration during abstinence on the expression of proinflammatory cytokines, markers of oxidative stress in the brain, and the activation of microglia. We aimed to contribute to a deeper understanding of the potential therapeutic advantages that fenofibrate might offer in the management of alcohol use disorder and the corresponding neurobiological alterations.

## 2. Materials and Methods

### 2.1. Animals and Treatments

High-drinker UChB (University of Chile Bibulous) rats derived from the Wistar strain and bred selectively for their high alcohol intake [38] were used. Two-month-old female rats were housed in individual cages in temperature-controlled rooms under a regular 12-hour light/12-hour dark cycle (a total of 30 animals). For 46 days, rats were offered a free choice between 10% (*v*/*v*) ethanol solution and water. On day 47, rats were allowed concurrent three-bottle choice access to 10% and 20% (*v*/*v*) ethanol solutions and water for 14 additional days. This protocol was routinely adopted by our group, since, in a previous study, we observed that simultaneous access to 10% and 20% ethanol allowed the detection of a more marked effect in relapse-type alcohol consumption [39]. Food (Mardones rat formula, Alimentos Cisternas, Santiago, Chile) and water were always provided ad libitum, and the volume of water and ethanol was recorded daily. After these 60 days of alcohol consumption, the weight of the animals averaged 242.1 ± 18.9 g. The decision to utilize female rats in this study stems from compelling evidence in specific rat lines, such as high-alcohol-drinking-2 (HAD-2), and Sardinian alcohol-preferring (sP) lines, which were selectively bred for their elevated ethanol consumption. In these lines, females consistently demonstrated higher levels of ethanol consumption compared to males [40,41]. This pattern is found as well in the UChB rat line (unpublished results). Furthermore, the choice of female rats is supported by their relatively more stable body weight during the 2–3-month duration of the experiments conducted in this study.

After 60 days of continuous free choice between ethanol and water, animals were divided into 4 groups (n = 7 for groups I and IV, n = 8 for groups II and III, see below) and deprived of ethanol access for 14 days, keeping water and food consumption ad libitum. In the last five days of ethanol withdrawal, the four groups were, respectively, administered a daily dose of: Group I, Vehicle control: dimethyl sulfoxide (DMSO), i.p., plus water by gavage, which are the vehicles of GW6471 and of fenofibrate, respectively; Group II, Fenofibrate: micronized fenofibrate (Fibronil^®^, Royal Pharma, Santiago, Chile) 50 mg/kg/day per gavage, re-suspended in water (1 mL per each 150 g of body weight) [27] plus DMSO i.p. vehicle; Group III, Fenofibrate: 50 mg/kg/day + GW6471 1 mg/kg/day i.p. (a PPAR-α specific antagonist, MyBioSource, San Diego, CA, USA), dissolved in DMSO (0.1 mL per 100 g body weight) [42]; and Group IV, GW6471: 1 mg/kg/day i.p. plus water vehicle per gavage.

On day 75, after finishing the 14 days of abstinence (which included the 5 days of drug treatment), the animals were offered the 10% and 20% (*v*/*v*) ethanol solutions again for just 1 h, and the consumed volume was recorded.

Immediately after recording alcohol consumption during 1 h re-access, the animals were anesthetized with ketamine/xylazine (10:1 mg/kg of body weight, i.p.) and immediately decapitated to obtain brains samples.

### 2.2. Quantification of the Expression of Proinflammatory Cytokines and Oxidative Stress Markers

From one of the cerebral hemispheres, hippocampus, nucleus accumbens, and prefrontal cortex tissues were extracted and homogenized in RNA-Solv^®^ Reagent (Omega Bio-tek, Inc., Norcross, GA, USA) with a mini Potter–Elvehjem pestle (Sigma-Aldrich, St. Louis, MO, USA). Total RNA was purified according to the manufacturer’s instructions. For RT-qPCR analysis, 300 ng of RNA was treated with DNase I (New England Biolabs, Ipswich, MA, USA) and subjected to amplification by RT-qPCR using the following primers: TNF-α (forward) CAGCCGATTTGCCACTTCATA, TNF-α (reverse) TCCTTAGGGCAAGGGCTCTT, IL1-β (forward) AGGCTTCCTTGTGCAAGTGT, IL1-β (reverse) TGTCGAGATGCTGCTGTGAG, IL6 (forward) CCCAACTTCCAATGCTCTCCTAATG, IL6 (reverse) GCACACTAGGTTTGCCGAGTAGACC, β-Actin (forward) CTTGCAGCTCCTCCGTCGCC β-Actin (reverse) CTTGCTCTGGGCCTCGTCGC, HO-1 (forward) TCGACAACCCCACCAAGTTC, HO-1 (reverse) AGGTAGTATCTTGAACCAGGCT.

The corresponding GenBank accession numbers are: *TNF-α* X66539.1; *IL1-β* NM_031512.2; *IL6* NM_012589.2; *β-Actin* NM_031144.3; HO-1 NM_012580.2.

### 2.3. Determination of Oxidized Glutathione (GSSG) and Reduced Glutathione (GSH) Levels

A portion of the hippocampus was reserved for the determination of oxidized glutathione (GSSG) and reduced glutathione (GSH) levels using the GSH/GSSG Ratio Detection Assay Kit II (Abcam, Boston, MA, USA), following the manufacturer’s instructions. Briefly, tissue samples were homogenized in ice-cold PBS/0.5% NP-40 with a mini Potter–Elvehjem pestle ((Sigma-Aldrich, St. Louis, MO, USA).), centrifuged for cell debris removal, and deproteinized to remove enzymes that could potentially metabolize glutathione (Deproteinizing Sample Preparation Kit—TCA, Abcam, Boston, MA, USA).

### 2.4. Determination of Microglia Immunoreactivity

The other cerebral hemisphere was used for immunofluorescence against the microglial marker ionized calcium-binding adaptor molecule 1 (Iba-1, 019-19741, Wako Chemicals, Richmond, VA, USA, 1:400 dilution) in coronal cryosections of the hippocampus (30 µm thick) as previously reported [43]. Nuclei were labeled with 4,6 diamino-2-phenylindol (DAPI), 0.02 M; 0.0125 mg/mL for nuclear labelling (Invitrogen, Carlsbad, CA, USA). Microphotographs were taken from the stratum radiatum of the hippocampus using a confocal microscope (Zeiss, LMS700, Oberkochen, Germany). The area analyzed for each stack was 0.04 mm^2^, and the thickness (*z*-axis) was measured for each case. The density of Iba-1-positive microglial cells was assessed using FIJI Image J analysis software (https://imagej.net/ij/, accessed on 6 September 2023) as previously reported [43].

### 2.5. Statistical Analyses

Statistical analyses were performed using GraphPad Prism 8. Data are expressed as means ± SEM. One-way or two-way ANOVA followed by Tukey’s post hoc analysis was used. A level of *p* < 0.05 was considered for statistical significance.

## 3. Results

### 3.1. Effect of Fenofibrate on Relapse-like Alcohol Consumption

**Figure 1:**
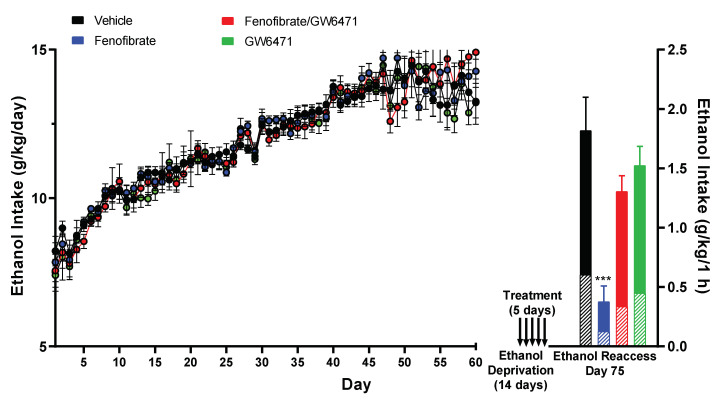
Fenofibrate administration during the ethanol withdrawal stage reduces alcohol relapse-like drinking. Following 46 days of chronic 10% (*v*/*v*) ethanol intake and then concurrent three-bottle choice access to 10% and 20% (*v*/*v*) ethanol solutions and water for 14 additional days, rats were deprived of ethanol for 14 days. In the last 5 days of deprivation, 4 groups were, respectively, treated with a daily dose of: (i) vehicle control (DMSO + water); (ii) fenofibrate 50 mg/kg/day + DMSO; (iii) fenofibrate 50 mg/kg/day plus GW6471 1 mg/kg/day; and (iv) GW6471 1 mg/kg/day + water. The day after finishing the 14 days of abstinence (which includes the 5 days of drug treatment), the animals were offered the 10% and 20% (*v*/*v*) ethanol solutions again for just 1 h, and the consumed volume was recorded. Hatched pattern in bars represents the amount of 20% ethanol ingested, while the filled pattern represents the amount of 10% ethanol; the total height of the bars and the standard errors shown correspond to the total amounts of alcohol consumed. Data are presented as mean ± SEM, n = 7–8 rats per experimental group. *** = *p* < 0.001 vs. Vehicle and Fenofibrate/GW6471 groups. ANOVA followed by Tukey’s test for multiple comparisons.

Over a span of 60 days, voluntary alcohol consumption was tracked within the four experimental groups. As shown in Figure 1 (left side), alcohol consumption increased from 7.6 ± 1.5 g/kg/day on day 1 (10% ethanol solution) until stabilizing at 13.86 ± 1.19 g/kg/day between days 47 and 60 (10% plus 20% ethanol solutions). Of this cumulative ethanol consumption, an average of 65% was attributed to the 10% ethanol solution bottle, while the remaining 35% was derived from the 20% ethanol solution bottle (data not presented in Figure 1 left). No significant differences were detected among the four groups (ANOVA F_(3,1586)_ = 1.023; *p* = 0.3816).

Following this period, the animals were deprived of alcohol for 14 days. In the last five days of the withdrawal period, the four groups were, respectively, treated with: (i) vehicles (DMSO i.p. and water gavage); (ii) fenofibrate 50 mg/kg/day per gavage + DMSO, (iii) fenofibrate 50 mg/kg/day by gavage plus GW6471 1 mg/kg/day i.p. (a PPAR-α specific antagonist); (iv) GW6471 1 mg/kg/day i.p. + water. GW6471 is a specific PPAR-α antagonist, with capacity to counteract the protective effects of PPAR-α on neuroinflammation [44]. As shown in Figure 1 (right side), the administration of fenofibrate in the withdrawal stage produced an 80% decrease in total voluntary alcohol consumption during the sole hour of re-access to alcohol. This reduction was markedly different from the control group, which solely received the vehicles (0.38 ± 0.13 g/kg/1 h vs. 1.82 ± 0.28 g/kg/1 h; ANOVA F_(3,26)_ = 15.47; *p* < 0.001). When fenofibrate was administered simultaneously with GW6471, the decrease in alcohol consumption during relapse was abrogated, since no significant differences were observed when compared to the control group that solely received the vehicles (1.31 ± 0.13 g/kg/1 h vs. 1.82 ± 0.28 g/kg/1 h; ANOVA *p* = 0.2030). Similarly, the administration of GW6471 alone also yielded no significant effect on alcohol consumption when compared to the control group (1.53 ± 0.16 g/kg/1 h vs. 1.82 ± 0.28 g/kg/1 h; ANOVA *p* = 0.6788). These findings provide strong evidence that the effect of fenofibrate on alcohol consumption during relapse is specifically due to PPAR-α activation.

Regardless of the total amount of alcohol ingested among the four groups, the ratio between 10% ethanol and 20% ethanol consumption during relapse remained statistically unchanged from that observed during the chronic consumption stage (vehicle group: 67%/33%; fenofibrate group: 66%/34%, fenofibrate+GW6471 group: 70%/30%, GW6471 group: 70%/30%), indicating that the different treatments did not change the preference for ethanol concentration in the ingested solution.

### 3.2. Effect of Fenofibrate on Ethanol-Induced Expression of Proinflammatory Cytokines and an Oxidative Stress Marker

In a previous study, we had reported the effectiveness of fenofibrate in reversing ethanol-induced increase in GFAP expression, and NF-κB activation in the hippocampus, prefrontal cortex, and the hypothalamus [37]. These findings align with fenofibrate´s propriety in inhibiting neuroinflammation in contexts beyond alcohol exposure, including aging, ischemia/reperfusion injury, and traumatic brain injury [32,33,34,35,36]. In our current investigation, we explored the ability of fenofibrate to counteract an increase in proinflammatory cytokines and a marker of oxidative stress, both induced by alcohol consumption. To quantify these effects, the expression of three well-known proinflammatory cytokines (*TNF-α, IL1-β*, and *IL-6*) as well as the expression of heme oxygenase-1 (*HO-1*), a gene that is induced in response to oxidative stress [44] was quantified by RT-qPCR in the hippocampus, nucleus accumbens (NAcc), and prefrontal cortex (PFC).

**Figure 2:**
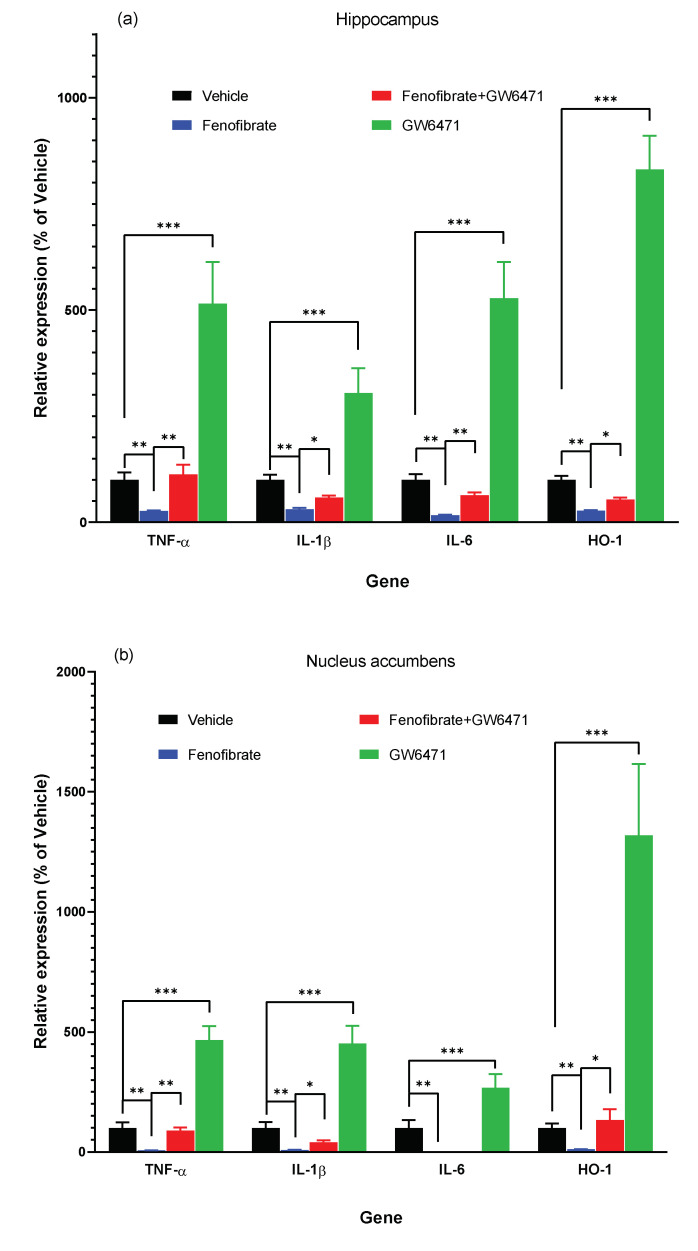

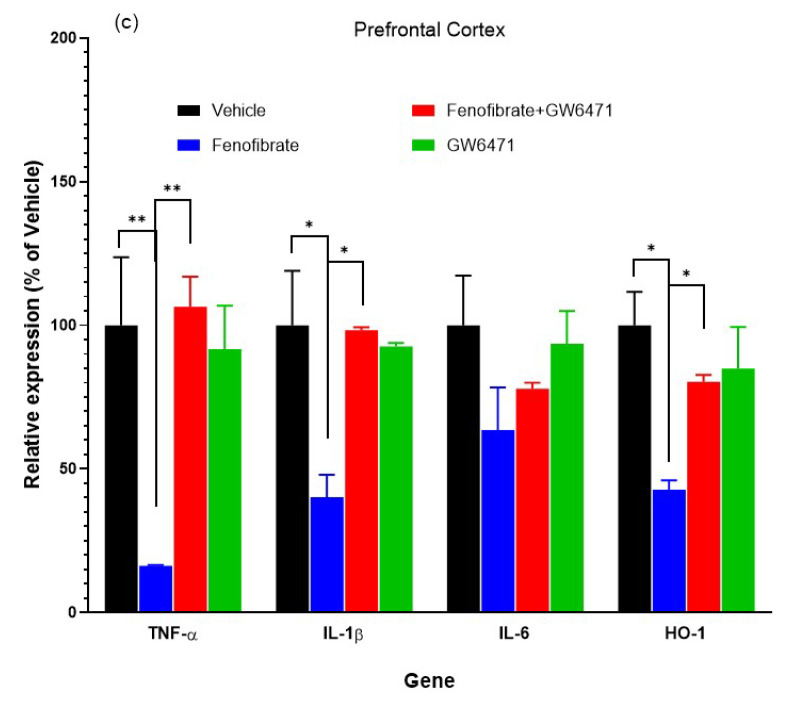
Fenofibrate administration during the ethanol withdrawal stage reduces the expression of proinflammatory cytokines and the marker of oxidative stress. Following the experiment shown in Figure 1, the expression *of TNF-α, IL-1β, IL-6*, and *HO-1* in the hippocampus (**a**), NAcc (**b**), and PFC (**c**) was analyzed by RT-qPCR in the 4 groups of animals, treated, respectively, with: (i) vehicle control (DMSO + water); (ii) fenofibrate 50 mg/kg/day + DMSO; (iii) fenofibrate 50 mg/kg/day plus GW6471 1 mg/kg/day; and (iv) GW6471 1 mg/kg/day + water. The graphs show the levels of gene expression as percentages of their vehicle-administered controls, normalized by the levels of expression of *ß-actin*. Data are presented as mean ± SEM, n = 7–8 rats per experimental group. * = *p* < 0.05, ** = *p* < 0.01, *** = *p* < 0.001 between the indicated groups. ANOVA followed by Tukey’s test for multiple comparisons.

As shown in Figure 2a–c, fenofibrate was able to decrease mRNA *TNF-α* expression in the hippocampus (fenofibrate vs. vehicle: 26.7 ± 1.5% vs. 100.0 ± 17.8%; ANOVA F_(3,26)_ = 28.0; *p* < 0.01), NAcc (6.2 ± 0.9% vs. 100.0 ± 23.7%; ANOVA F_(3,26)_ = 80.2; *p* < 0.01), and PFC (16.3 ± 0.3% vs. 100.0 ± 23.7%; ANOVA F_(3,26)_ = 12.0; *p* < 0.01). Fenofibrate was also able to decrease the mRNA expression of *IL-β* in the hippocampus (fenofibrate vs. vehicle: 30.3 ± 3.5% vs. 100.0 ± 11.5%; ANOVA F_(3,26)_ = 32.9; *p* < 0.01), NAcc (7.8 ± 1.3% vs. 100.0 ± 25.1%; ANOVA F_(3,26)_ = 53.0; *p* < 0.01), and PFC (40.2 ± 7.8% vs. 100.0 ± 18.9%; ANOVA F_(3,26)_ = 11.0; *p* < 0.05). Similar results were observed regarding the mRNA expression of *IL-6*, since fenofibrate showed effects in the hippocampus (fenofibrate vs. vehicle: 16.8 ± 1.1% vs. 100.0 ± 13.2%; ANOVA F_(3,26)_ = 46.2; *p* < 0.01) and NAcc (0.0 ± 0.0% vs. 100.0 ± 33.1%; ANOVA F_(3,26)_ = 12.0; *p* < 0.01). In the case of PFC, although there was a decrease, it was, albeit marginally, not statistically significant (63.6 ± 15.0% vs. 100.0 ± 17.3%; ANOVA F_(3,26)_ = 2.6; *p* = 0.07). Regarding the oxidative stress marker gene *HO-1*, the administration of fenofibrate showed marked effects in reducing its expression in the hippocampus (fenofibrate vs. vehicle: 27.7 ± 1.3% vs. 100.0 ± 9.4%; ANOVA F_(3,26)_ = 18.0; *p* < 0.01), NAcc (11.0 ± 0.6% vs. 100.0 ± 19.2%; ANOVA F_(3,26)_ = 43.7; *p* < 0.01), and PFC (42.8 ± 3.2% vs. 100.0 ± 11.7%; ANOVA F_(3,26)_ = 9.0; *p* < 0.05), which would indicate its ability to reduce not only neuroinflammation but also ethanol-induced oxidative stress in these brain areas.

The co-administration of the antagonist GW6471 and fenofibrate abrogated the effect of the latter as in alcohol consumption during relapse (Figure 1), further reinforcing the notion that the reduced expression of proinflammatory cytokines and the oxidative stress marker is explicitly attributed to PPAR-α activation (Figure 2). [Fenofibrate + GW6471 vs. fenofibrate, *TNF-α*: hippocampus 113.0 ± 22.4% vs. 26.7 ± 1.5% ANOVA F_(3,26)_ = 28.0; *p* < 0.01; NAcc 88.7 ± 13.2% vs. 6.2 ± 0.9% ANOVA F_(3,26)_ = 80.2; *p* < 0.01; PFC 106.5 ± 10.5% vs. 16.3 ± 0.3% ANOVA F_(3,26)_ = 12.0; *p* < 0.01; *IL-1β*: hippocampus 58.5 ± 4.7% vs. 30.3 ± 3.5% ANOVA F_(3,26)_ = 32.9; *p* < 0.05; NAcc (40.9 ± 7.4% vs. 7.8 ± 1.3%; ANOVA F_(3,26)_ = 53.0; *p* < 0.05); PFC 98.3 ± 1.0% vs. 40.2 ± 7.8%; ANOVA F_(3,26)_ = 11.0; *p* < 0.05; *IL-6*: hippocampus 63.2 ± 7.2% vs. 16.8 ± 1.1% ANOVA F_(3,26)_ = 46.2; *p* < 0.01; NAcc 0.0 ± 0.0% vs. 0.0 ± 0.0% ANOVA F_(3,26)_ = 12.0, n.s.; PFC 77.9 ± 2.1% vs. 63.6 ± 15.0% ANOVA F_(3,26)_ = 2.6; *p* = 0.07; *HO-1*: hippocampus 53.7 ± 4.4% vs. 27.7 ± 1.3%; ANOVA F_(3,26)_ = 18.0; *p* < 0.05; NAcc (132.8 ± 45.9% vs. 11.0 ± 0.6%; ANOVA F_(3,26)_ = 43.7; *p* < 0.05; PFC 80.3 ± 2.5% vs. 42.8 ± 3.2% ANOVA F_(3,26)_ = 9.0; *p* < 0.05].

Notably, in the group administered only with the antagonist GW6471, a marked increase in all proinflammatory cytokines evaluated and in the marker of oxidative stress was detected, approximately between 3- and 13-fold in the hippocampus and NAcc (Figure 2) [GW6471 vs. vehicle, *TNF-α*: hippocampus 515.3 ± 98.1% vs. 100.0 ± 17.8% ANOVA F_(3,26)_ = 28.0; *p* < 0.001; NAcc 466.7 ± 57.9% vs. 100.0 ± 23.7% ANOVA F_(3,26)_ = 80.1; *p* < 0.001; *IL-1β*: hippocampus 304.5 ± 58.6% vs. 100.0 ± 11.5%; ANOVA F_(3,26)_ = 32.9; *p* < 0.001; NAcc (452.9 ± 73.7% vs. 100.0 ± 25.1%; ANOVA F_(3,26)_ = 53.0; *p* < 0.001); *IL-6*: hippocampus 528.0 ± 85.5% vs. 100.0 ± 13.2%; ANOVA F_(3,26)_ = 46.2; *p* < 0.01; NAcc 267.2 ± 57.4% vs. 100.0 ± 33.1%; ANOVA F_(3,26)_ = 12.0; *p* < 0.001; *HO-1*: hippocampus 831.0 ± 79.3% vs. 100.0 ± 9.4%; ANOVA F_(3,26)_ = 18.0; *p* < 0.001; NAcc (1318.7 ± 296.5% vs. 100.0 ± 19.2%; ANOVA F_(3,26)_ = 43.7; *p* < 0.001)].

### 3.3. Effect of Fenofibrate on the Levels of the Antioxidant Glutathione

**Figure 3:**
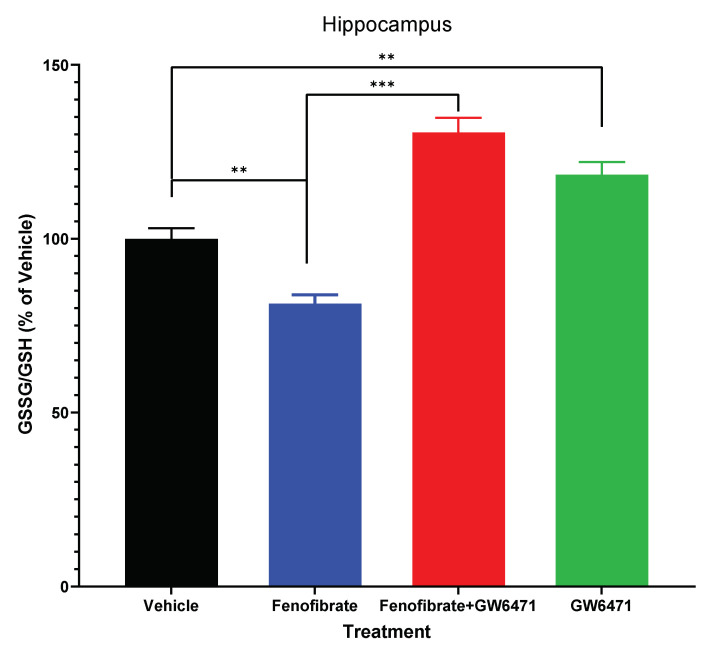
The administration of fenofibrate reduces the oxidative stress induced by ethanol. Oxidized glutathione (GSSG) and reduced glutathione (GSH) levels were determined in the hippocampus of the 4 groups of treated animals. The graph shows the GSSG/GSH ratios as percentages of their vehicle-administered controls. Data are presented as mean ± SEM, n = 7–8 rats per experimental group. ** = *p* < 0.01 and *** = *p* < 0.001 between the indicated groups. ANOVA followed by Tukey’s test as a post hoc.

Figure 3 shows that the administration of fenofibrate decreased the oxidized glutathione/reduced the glutathione ratio (GSSG/GSH) in the hippocampus (fenofibrate vs. vehicle: 81.4 ± 2.5% vs. 100.0 ± 3.1%, ANOVA F_(3,26)_ = 41.3; *p* < 0.01). Like what was observed with the expression of proinflammatory cytokines and HO-1 (Figure 2), co-administration of the antagonist GW6471 decreased the protective effect of fenofibrate against oxidative stress (fenofibrate+GW6471 vs. fenofibrate: 130.6 ± 4.2% vs. 100.0 ± 3.1%, ANOVA F_(3,26)_ = 41.3; *p* < 0.001). Furthermore, administration of GW6471 alone produced per se an increase in oxidative stress in the hippocampus (GW6471 vs. vehicle: 118.4 ± 3.7% vs. 100.0 ± 3.1% ANOVA F_(3,26)_ = 41.3; *p* < 0.01). These findings indicate that fenofibrate can reduce not only neuroinflammation, but also oxidative stress levels in this brain area, which is consistent with the decrease in HO-1 expression levels (Figure 2a), together with the decrease in the relapse-like alcohol intake (Figure 1).

Similar to what was observed with the expression of proinflammatory cytokines and with the oxidative stress-induced gene (HO-1) in the hippocampus and NAcc (Figure 2), the administration of the antagonist GW6471, either in conjunction with fenofibrate or alone, produced a 30.6% and 18.4% increase in oxidative stress levels in the hippocampus, respectively (fenofibrate+GW6471 vs. vehicle: 130.6 ± 4.2% vs. 100.0 ± 3.1%, GW6471 vs. vehicle: 118.4 ± 3.6% vs. 100.0 ± 3.1%, ANOVA F_(3,26)_ = 41.3; *p* < 0.01).

### 3.4. Effect of Fenofibrate on Ethanol-Induced Microglial Reactivity

**Figure 4:**
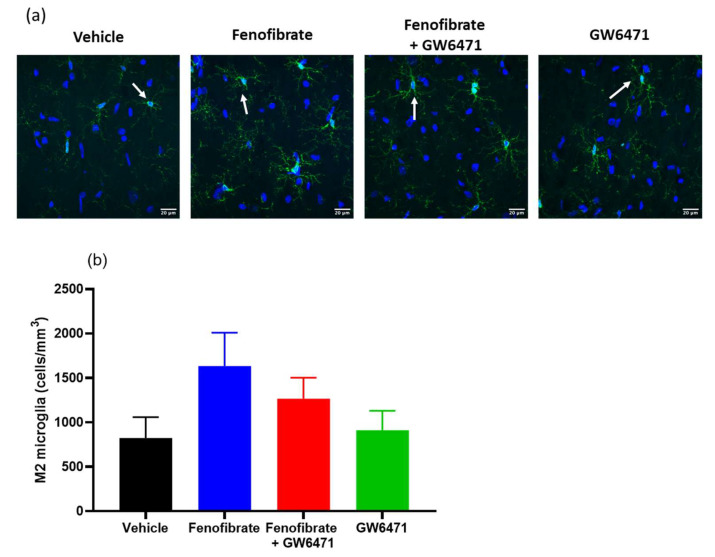
Effect of fenofibrate administration on microglial reactivity. The cells with the M2 (anti-inflammatory) phenotype were quantified in the hippocampus of the 4 groups of treated animals. (**a**) Representative microphotograph of microglia immunofluorescence (IBA-1 immunoreactivity: green; DAPI: blue) from the four experimental groups. Scale bar 20 μm. The arrows show an example of the morphology of the cells that were counted. (**b**) Densitometric analysis from Iba-1-positive cells/mm^3^ present in 3 hippocampal slices from each animal. Data are presented as mean ± SEM, n = 7–8 rats per experimental group. ANOVA followed by Tukey’s test as a post hoc.

Regarding microglia reactivity, while not reaching statistical significance, fenofibrate showed a trend towards doubling the number of cells displaying an anti-inflammatory morphology (M2) in comparison to the vehicle group (Figure 4) (fenofibrate vs. vehicle: 1633 ± 375 vs. 823 ± 234, ANOVA F_(3,86)_ = 1.76; *p* = 0.16). In addition, the co-administration of the antagonist GW6471 together with fenofibrate attenuated the effect of the latter on the increase in the number of M2 microglial cells (fenofibrate+GW6471 vs. fenofibrate: 1266 ± 235 vs. 1633 ± 375, ANOVA F_(3,86)_ = 1.76; *p* = 0.16).

**Figure 5:**
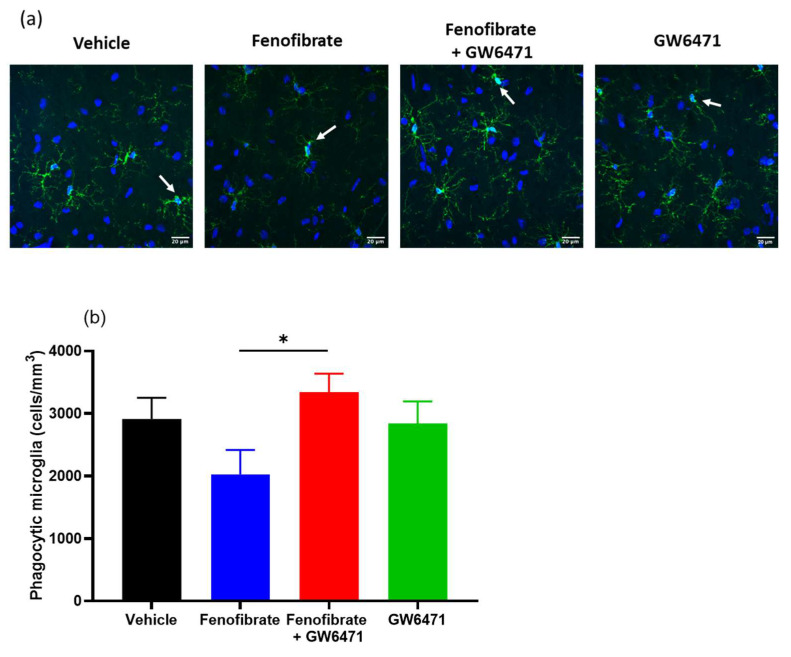
Effect of fenofibrate administration on microglial reactivity. The microglial cells with the phagocytic phenotype were quantified in the hippocampus of the 4 groups of treated animals. (**a**) Microglia immunofluorescence (IBA-1 immunoreactivity: green; DAPI: blue). Scale bar 20 μm. The arrows show examples of the morphology of the microglial cells in phagocytic process. (**b**) Densitometric analysis of microglial cells in the phagocytic process (phagocytic pouches). Data are presented as mean ± SEM, n = 7–8 rats per experimental group. * = *p* < 0.05, between the indicated groups. ANOVA followed by Tukey’s test as a post hoc.

We also quantified phagocytic microglial cells in the hippocampus. Phagocytic microglia are characterized by rounded macrophage-like morphology with no or few processes and are associated with maximal proinflammatory activation and oxidative-free radicals [11]. The group of animals treated with fenofibrate showed a decrease of 30.4% with respect to the control group, although the difference was statistically significant only when compared with the fenofibrate+GW6471 group (ANOVA F_(3,86)_ = 2.66 *p* < 0.05) (Figure 5).

## 4. Discussion

Due to the marked effect of fenofibrate in decreasing post-withdrawal relapse-type alcohol consumption (Figure 1), it is reasonable to consider whether this could be attributed to effects that extend beyond the inhibition of the drinking reflex, e.g., producing memory and/or spatial orientation disorders, mobility impairment, sedation, anxiety, depression, etc. Although in this work we did not carry out behavioral or motor studies, there are studies by other authors that have shown that fenofibrate decreases the motivation of animals to obtain ethanol (operant self-administration) but not to self-administer sucrose [30], which could indicate that there are no effects on memory or spatial orientation, or other effects such as depression (depressed animals decrease their sucrose intake) or sedation. In another study, Blednov et al. [29] reported that fenofibrate did not alter preference for saccharin, nor motor response to novelty, reduced duration of ethanol-induced loss of righting reflex, and increased EtOH-induced conditioned taste aversion. That is, fenofibrate per se does not produce motor effects that could explain its effect in reducing alcohol consumption.

In addition to the decrease in relapse-type alcohol consumption, fenofibrate showed effects in a reduction in the expression of proinflammatory cytokines and of a gene that is induced by oxidative stress (*HO-1*), in the brain (Figure 2). Although the effects of PPAR-α in mitigating ethanol-induced brain oxidative stress had not previously been explored, its neuroprotective capabilities have been reported in distinct models [45], including traumatic brain injury [31], transient cerebral ischemia/reperfusion [34], and Alzheimer’s disease [46]. Collino et al. [34] reported that the administration of a synthetic PPAR-α agonist (WY14643) reduced *HO-1* expression induced by ischemia/reperfusion brain injury. Additionally, oleoylethanolamide (a physiological PPAR-α agonist) administration in a model of chemically induced degeneration of substantia nigra dopamine neurons, led to a decrease in the number of HO-1 immunoreactive cells in the striatum when compared to untreated animals [47]. Nevertheless, there are also several reports where the expression of *HO-1* increases due to treatment with anti-inflammatory agents. It has been found that administration of the antioxidant N-acetylcysteine to UChB rats with chronic alcohol consumption was linked to increased *HO-1* expression [48]. The discordant findings concerning the decrease or increase in *HO-1* expression following particular anti-inflammatory treatments could be attributed to various factors, including the animal model employed, the nature of the anti-inflammatory drug, the route and duration of administration, and the timing of *HO-1* expression evaluation post-treatment. Specifically, in the UChB rat model, the distinction between the study conducted by Quintanilla et al. [48] and our findings lies in the fact that N-acetylcysteine was administered over a 14-day span during the period of alcohol consumption. In contrast, fenofibrate was administered for only 5 days during the withdrawal period. Nonetheless, since the induction of *HO-1* is mediated by ROS production [44] it remains unclear whether the reduction in *HO-1* expression is a direct outcome of fenofibrate or a consequence of diminished oxidative stress levels in rats to which fenofibrate was administered after chronic alcohol consumption.

Remarkably, the group that received only the antagonist GW6471 exhibited a considerable rise in all assessed proinflammatory cytokines, along with an elevated expression of *HO-1*, demonstrating an increase ranging from approximately 3- to 13-fold in the hippocampus and NAcc (Figure 2). The substantial increases induced by the administration of GW6471 alone (Figure 2) do not correlate with an increase in alcohol consumption of this group during the re-access stage (Figure 1). This discrepancy likely arises because the amount of alcohol that UChB rats can drink in 1 h has already reached its ceiling of ~1.5–1.8 g/kg/1 h, as evidenced in other reports from our group [43]. PPAR-α is not only activated by drugs of the fibrate family; it also responds to endogenous agonists such as palmitoylethanolamide [49] and oleoylethanolamide [50]. Furthermore, the anti-inflammatory and antioxidant actions of these agonists in the brain have been demonstrated [49,51,52]. In a similar way to fibrates, it has been reported that these endogenous agonists exert effects in reducing voluntary alcohol consumption in animals [53,54]. Thus, it is possible that the administration of GW6471 alone produced a degree of PPAR-α inhibition that prevented its activation by endogenous agonists, consequently inhibiting its anti-inflammatory and antioxidant functions against the deleterious effects of ethanol. The minor response within the PFC in comparison to the hippocampus and NAcc could be attributed to the fact that PFC has been observed to display a comparative lower response to alcohol administration [37]. In agreement, Kane et al. [55] reported that ethanol administration fails to increase the expression of pro-inflammatory cytokines like chemokine C-C motif ligand 2 (CCL2), IL-6, and TNF-α in the mouse cerebral cortex.

Additionally, fenofibrate showed effects in reducing oxidative stress in the hippocampus, measured as the GSSG/GSH ratio. In several reports, the reduction in relapse-like alcohol intake has been related to a decrease in cerebral oxidative stress through the administration of N-acetylcysteine (a precursor for the cellular synthesis of glutathione) [48], ibudilast (an anti-inflammatory phosphodiesterase inhibitor) [56], and mesenchymal stem cells and their secreted products known for potent anti-inflammatory actions [43]. In our study, it was found that the administration of fenofibrate produced approximately 20% reduction in the GSSG/GSH ratio. Quintanilla et al. reported a decrease of 70% in the GSSG/GSH ratio by N-acetylcysteine [48] and 45% due to the secretome derived from mesenchymal stem cells [43]. The significant reduction achieved by N-acetylcysteine may be attributed to its direct function as a precursor for the synthesis of glutathione, whereas the effect of fenofibrate seems to be indirect. Regarding the difference obtained for secretome derived from mesenchymal stem cells, this was administered in 3 doses spread over 18 days of treatment [43], a longer treatment compared to fenofibrate (5 days). Moreover, the mechanisms underlying these effects may not necessarily be identical. In agreement with our results, in a model of oxidative stress induced by valproic acid, fenofibrate decreased 33% the levels of GSH [57]. The administration of the GW6471 antagonist, whether in combination with fenofibrate or alone, resulted in a rise in oxidative stress levels in the hippocampus (Figure 3). As previously discussed in relation to Figure 2, it is plausible to consider that the administration of GW6471 resulted in a substantial inhibition of PPAR-α, preventing its activation by endogenous agonists and consequently inhibiting its antioxidant activity against the detrimental effects of ethanol.

We also studied the properties of fenofibrate on the reactivity of microglia in the hippocampus, as a parameter to evaluate its protective effects against ethanol-induced neuroinflammation. We observed a tendency to increase the number of microglia with anti-inflammatory phenotype (M2) (Figure 4) and to reduce phagocytic microglial cells (Figure 5). In contrast to the effects observed on alcohol consumption during relapse (Figure 1), the expression of proinflammatory cytokines (Figure 2) and oxidative stress levels (Figure 3), the relatively short treatment period of five days with fenofibrate might not have been sufficient to elicit a more pronounced impact on the microglial cell phenotype. In future experiments, we plan to evaluate the effect of longer treatments with fenofibrate. According to this idea, Quintanilla et al. [48] and Ezquer et al. [43] were able to observe differences in microglia cells in the hippocampus of UChB rats that had consumed alcohol and were then treated with N-acetylcysteine or mesenchymal stem cell-derived secretome, respectively, by employing extended treatment periods.

One of the considerations that we must make as caveat of the current study is that we did not include groups of rats that had not drunk alcohol. In several publications of our group (please see references [43,48], as examples), we have reported that this scheme of alcohol administration and subsequent withdrawal produces a neuroinflammation response and oxidative stress in the brain of UChB drinking rats compared to alcohol-naïve rats. The objective of the current work was to demonstrate that, if fenofibrate was administered during the withdrawal stage to rats that had previously voluntarily drunk alcohol chronically, it would decrease voluntary alcohol consumption when it was offered again to the animals (model of relapse-type drinking), and that these effects are mediated by PPAR-α activation. Thus, it would have been somewhat difficult to include in this scheme a group of alcohol-naïve animals, since we would not have been able to assess relapse in animals that had never drunk. Regarding the effect of fenofibrate on markers of neuroinflammation and oxidative stress when administered to naïve animals, there are some reports in the literature. For example, Barbiero et al. [58] reported that fenofibrate per se does not produce changes in oxidative stress (GSH levels, superoxide dismutase, lipid peroxidation) in the striatum of rats. In other studies, the administration of fenofibrate to control rats did not produce changes in the expression of TNF-α, IL-1β, or IL-6 in the spinal cords of mice [59] nor in inflammatory markers such as prostaglandin D2, thromboxane, arachidonic acid, cyclooxygenase-2, and TNF-α in rat primary astrocyte cultures [60]. For their part, Mirza and Sharma [57] reported that fenofibrate did not change the levels of GSH, IL-6, and TNF-α in the cerebellum, brainstem, and prefrontal cortex of rats. Based on this background, we believe that the administration of fenofibrate per se should not produce alterations in the proinflammatory markers when administered to alcohol-naïve animals.

Another limitation that we must consider is regarding the use of only female animals in this study. As we have stated above, UChB females, as well as females from other lines selected to drink alcohol, present a higher consumption compared to males. However, we cannot rule out that the response to fenofibrate could have different characteristics between male and female UChB rats. In this sense, Blednov et al. [61] reported that male and female mice that drank alcohol respond in different ways to treatment with PPAR agonists.

We are also aware that in these studies we do not include naive animals that have not been administered DMSO. In a recent systematic review, Dludla et al. [62] found that DMSO possesses essential antioxidant properties that are linked to its protective effect against oxidative damage. Interestingly, this antioxidant effect appears to be dose-dependent in vivo, since lower doses (1–3 g/kg) were the most effective in blocking oxidative stress-induced damage, mostly through a reduction in ROS, inhibition of lipid peroxidation, improving mitochondrial function, enhancement of intracellular antioxidants, and suppression of pro-inflammatory makers, in the different studies analyzed. On the other hand, high doses of DMSO have been shown to be toxic and induce oxidative stress and cellular damage [62]. In our studies, we administered a DMSO dose of 1 mL/kg—which is equivalent to 1.1 g/kg—that is, in the range of the lowest doses used in the literature. The possibility exists that the anti-inflammatory effects of DMSO might have overshadowed the anti-inflammatory and anti-oxidative stress effects of fenofibrate; however, we do not believe this would have been the case, as only animals receiving fenofibrate showed effects on downregulation of proinflammatory and oxidative stress cytokine genes, as well as a decreased GSSG/GSH ratio, while the four groups of rats indeed did receive DMSO.

## 5. Conclusions

Overall, our studies show that the administration of fenofibrate to rats during the withdrawal stage after chronic ethanol consumption decreases the severity of relapse when ethanol is offered again to the animals. This beneficial effect apparently is due to the capacity of fenofibrate to reduce ethanol-induced neuroinflammation. This was evident in the downregulation of proinflammatory cytokines and oxidative stress-induced genes in the brain, decreased oxidative stress (GSSG/GSH ratio), the decreased number of phagocytic microglial cells, and a trend to increase the number of anti-inflammatory microglia. These protective effects collectively could contribute to a reduction in ethanol craving, which is the main cause of relapse in patients undergoing detoxification. Our findings suggest that incorporating fenofibrate into the post-detoxification withdrawal phase could serve as a promising therapeutic approach to prevent or alleviate the severity of relapse. The ultimate goal of our research is to identify a novel and effective pharmacological treatment for AUD, based on a drug already approved for other clinical conditions. In this context, fenofibrate has been clinically used worldwide for decades, having received approval in Europe in the 1980s, and in USA since 1994 for the treating for dyslipidemia.

## Data Availability

We have no additional data available to share.
